# The Cyprus Herp Atlas: An initiative for systematic recording of amphibian and reptile occurrences in Cyprus

**DOI:** 10.3897/BDJ.11.e98142

**Published:** 2023-05-09

**Authors:** Savvas Zotos, Marilena Stamatiou, Ioannis N. Vogiatzakis

**Affiliations:** 1 Faculty of Pure and Applied Sciences, Open University of Cyprus, Latsia, Nicosia, Cyprus Faculty of Pure and Applied Sciences, Open University of Cyprus Latsia, Nicosia Cyprus; 2 Herpetological Society of Cyprus, Paphos, Cyprus Herpetological Society of Cyprus Paphos Cyprus

**Keywords:** citizen science, Cyprus, database, herpetofauna, repository

## Abstract

Even though the reptiles and amphibians of Cyprus are of scientific and conservation importance and although several books, guides and scientific reports have been published the past 30 years, there is a clear absence of a systematic recording and archiving scheme of all available data in a structural database. Towards this end, the Cyprus Herp (= reptiles and amphibians) Atlas has been developed. The Atlas constitutes the first effort to collect all existing locality data of the herpetofauna species of the island (i.e. scientific reports, books, journals, grey literature) in a single database and simultaneously promote a citizen-science approach in order to collect and constantly update the database with new records.

The website of the Atlas contains basic educational and informational material for the public, along with the visibility tool of the database in the form of occurrence maps, in 5 km x 5 km grid cells, openly available for download in kmz format. The Atlas is a powerful tool for citizens, scientists and decision-makers, aiming to contribute to the study and conservation of the reptile and amphibian species of Cyprus. In this short communication, we give details on the structure of the Atlas.

## Introduction

The island of Cyprus, the third largest island in the Mediterranean with an area of 9,251 km^2^, is situated in the eastern part of the globally-important Mediterranean Basin biodiversity hotspot (Myers et al. 2000). The island hosts a total of 23 native terrestrial herps (= reptiles and amphibians), which consist of eleven lizards, eight snakes, one terrapin, two frogs and one toad species. Amongst them, three lizards, one snake and one frog are endemic to the island, while two of the species (*Acanthodactylusschreiberi* Boettger, 1878 and *Hierophiscypriensis* (Schätti, 1985)), have been declared as Endangered (EN) by the International Union for Conservation of Nature (IUCN).

Although most of the herpetofauna species are protected under National law (Cyprus Parliament 2003), European legislation (European Council 1992) or international agreements (Bern Convention 1982), there is a great lack of information for many of them. Several studies have been conducted during the past 30 years, but they are mostly focused on the zoogeography of Cypriot herpetofauna (Böhme and Wiedl 1994, Sindaco et al. 2000, Göçmen and Böhme 2002, Baier et al. 2013), the phylogeography (geographic distributions of genealogical lineages) of selected species in conjunction with the separation of the island of Cyprus from the mainland (Poulakakis et al. 2013, Tamar et al. 2014, Tamar et al. 2015, Kornilios 2017, Karameta et al. 2022) and the distribution of endemic species (Blosat et al. 1996, Baier et al. 2014, Erotokritou and Vogiatzakis 2019, Zotos et al. 2021, Erotokritou et al. 2022, Zotos et al. 2022). Few studies have tried to assess population size and structure (Blosat 2002, Michaelides and Kati 2009), while only four PhD studies have tried to reveal biological and ecological parameters of selected species (Blosat 1998, Zotos 2014, Savvides 2018, Karameta 2018).

The extensive road network of the island has been identified as a major pressure to biodiversity (Zomeni and Vogiatzakis 2014) and its impact on herpetofauna is currently being investigated (Zotos and Vogiatzakis 2018, Zotos et al. 2018) using citizen science for data collection (www.cyroadkills.org). Furthermore, a number of conservation activities for the threatened reptiles of the island have been conducted as part of a series of European Funded conservation projects (e.g. COMANACY - LIFE04 NAT/CY/000013, ICOSTACY - LIFE09 NAT/CY/00247), while new activities are been scheduled as part of an ongoing LIFE IP project (PANDOTEIRA - LIFE18 IPE/CY/000006).

In 2003, Hans-Jorg Wiedl published a small booklet on the snakes of Cyprus (Wiedl 2003) while, in 2009, Baier, Sparrow and Wiedl developed the first comprehensive book on the amphibians and reptiles of Cyprus (Baier et al. 2009) that included a thorough and complete collection of all scientific knowledge at the time, followed by a second edition in 2013 (Baier et al. 2013). A layman book was also published in Greek by the Herpetological Society of Cyprus the following year (Nicolaou et al. 2014).

Despite all this work, there is a clear absence of a systematic recording and archiving of all available locality data in a structural database, while the rapidly growing field of citizen science and its ability to contribute to research and conservation (Tulloch et al. 2013, Brown and Williams 2018, MacPhail and Colla 2020) is not well explored and utilised. Only a handful of citizen science initiatives are currently running in Cyprus, including the Cyprus Roadkill Observation System, that uses citizen's report of Wildlife Vehicle Collision (WVCs) to assess the impact of road network on biodiversity (Zotos and Vogiatzakis 2018) and initiatives for charismatic animal species, such as birds (https://birdlifecyprus.org), butterflies (https://butterfly-monitoring.net/cyprus-bms) and dragonflies (Sparrow et al. 2021).

The Cyprus Herp Atlas attempts to fill this gap by collating all existing locality data of the herpetofauna of the island (i.e. scientific reports, books, journals, grey literature) in a single well-structured database with the parallel promotion of a citizen-science approach for the collection of new records and the constant update of the database. The structure of the Cyprus Herp Atlas and the approaches followed come to address major biodiversity monitoring needs as prioritised for Mediterranean islands (Peyton et al. 2022). Taking advantage of the friendly interface and the openly-available maps of the Cyprus Herp Atlas, we will further promote the involvement and engagement of young people, including students, recognising the importance fof citizen science.

The Cyprus Herp Atlas is an initiative of the Terrestrial Ecosystems Management Lab (TemLab) of the Open University of Cyprus (http://temlab.ouc.ac.cy) and the Herpetological Society of Cyprus (https://hscyprus.org). The effort commenced in early 2022, as there was no available centralised collection of species localities for the herpetofauna of the island, which made academic research and even wildlife conservation and management decisions overcomplicated and tedious. Without making use of the fullest possible collection of localities for each species, most, if not all, recent herpetological research on the island so far has been based on partial locational information. The Atlas is an invaluable resource for the conservation and management of the Cypriot herpetofauna on all possible levels.

## Description of the Atlas

**Figure 1.**
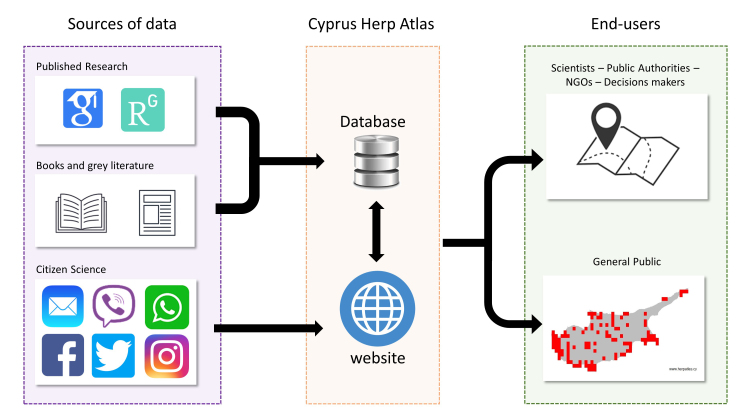
Interconnection between the features of the Cyprus Herp Atlas (i.e. Database and website) with the main sources of data and the main end-users.

The Atlas consists currently of the main database and a website interface (Fig. 1).

The Atlas’ database combines information from all possible sources: from past research and monitoring schemes providing geolocational information, existing literature such as books and journals, personal databases and citizen-science initiatives. The database includes primary Data that are the geospatial occurrence of species (latitude and longitude of occurrences) and Metadata that withholds other vital information, as described in the “Description of the database” section. The full database is available only upon request.

The website interface of the Atlas is used as a channel of communication, for both the reception and the provision of data, to scientists, decision-makers and the public. The website provides access to basic information on the biology and ecology of each species, as well as current occurrence maps in 5 km x 5 km grid cells that can be downloaded in kmz format. The website promotes citizen science by encouraging people to provide information on localities of reptiles and amphibians on the island. This citizen-science approach is reinforced by the activities of the Atlas in social media (e.g. Facebook, Twitter, Instagram).

Currently, the Atlas contains approximately 6,600 locality data from all 23 terrestrial reptiles and amphibians of Cyprus. From those data, 33% come from recent citizen science reports on the social media, citizen science initiatives (http://cyroadkills.org, http://herprepository.org) and public repositories (https://observation.org, https://www.inaturalist.org, https://naturgucker.de, https://www.hausdernatur.at) and can be considered as complete (data and metadata collected). In the case of public repositories, data were downloaded from www.gbif.org (GBIF.org 2022b, GBIF.org 2022a) and were cleaned before being transferred to the Atlas’ database. Data with invalid or poor coordinate accuracy (coordinate uncertainty greater than 500 metres) were removed. Nearly 43% of the data come from books, grey literature and public authorities reports and they bear partially incomplete metadata, while 25% have a total lack of metadata. New localities are consistently added to the database as the collection of published research and grey literature continues, while new data are being submitted by citizens.

The updating and management of the Atlas is conducted by a multidisciplinary team of scientists with expertise in relevant sciences including ecology, herpetology and geospatial analysis. The team members:

a) Conduct bibliographical research to identify published sightings;

b) Communicate with researchers who upkeep personal databases;

c) Collect data from citizens through social media and personal communication;

d) Assess, validate and organise all collected data, incorporating to and maintaining the database;

e) Retain communication channels with the public (i.e. answering questions and concerns, organising events) and promoting the citizen-science aspect of the Atlas.

It is important to acknowledge the laborious procedure of validating the received information, since, for cases of layman citizens and students, the team members need to review the provided information (mainly the pictures or videos provided), clarify unprovided metadata via personal communication and proceed to corrections, if needed, before incorporating to the Herp Atlas’ database.

## Description of the database

The database is currently archived in a comma-separated values (CSV) format, using Microsoft Excel software, in order to be easily uploaded and manipulated for spatial or statistical analysis in various software (e.g. ArcGIS, QGIS, R Studio). The spatial reference is kept in the World Geodetical System 1984 (WGS84).

The database has been designed to be easily updated, on a regular basis and easily manipulated, based on the needs of any researcher who might request it. The data are cohesive and easily searchable per species or source, while every observation takes up a single row and is coded with a reference ID number.

**Figure 2.**
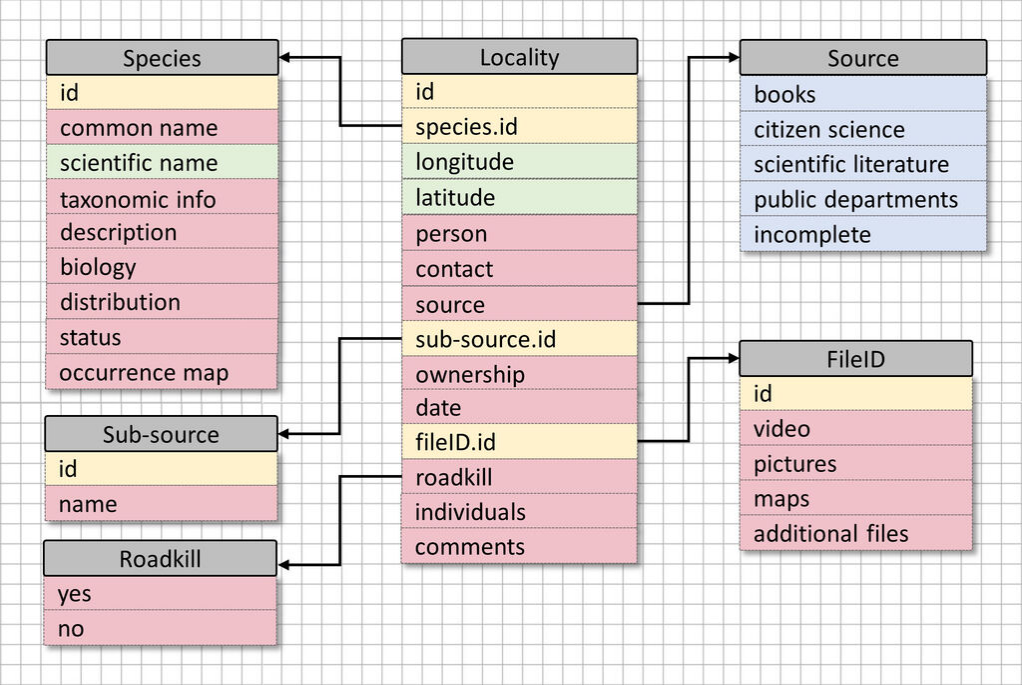
Database schema illustration holistically describing the data sources (blue), Data (green) and Metadata (pink) comprising the Cyprus Herp Atlas database.

Additional information, collected for each observation not able to be included in this simple CSV format (e.g. pictures, videos, kmz files, pdf files), is stored on external folders linked to the FileID field of the database. The database is regularly updated and maintained monthly. The newly-collected observations are assessed and added, while errors are checked and rectified. The structure of the database can be seen in Fig. 2, while more information on the Data and Metadata are presented in Table 1.

## Description of the website

The website of Cyprus Herp Atlas is accessible in Greek and English at https://www.herpatlas.cy. The website aims to be a user-friendly platform providing information and data on Cyprus’ herps and their occurrence on the island, simultaneously allowing citizens to contribute by providing their own information. The website has been created using google sites and it consists of six top-level pages, which are the following:

**Home page**: The home page includes simple information about this initiative and an introduction to the contents, sources and possible uses of the Atlas and the relevant database.

**General information**: General information on the reptiles (lizards, snakes, terrapins) and amphibian (anura) species of Cyprus. The page contains information related to different groups, families and species situated in Cyprus, their morphological and biological characteristics, along with their main ecological needs.

**Species**: The page contains four sub-pages, one for each category of herp in Cyprus (i.e. amphibians, lizards, snakes, terrapins). Each sub-page has a photographic catalogue of the relevant species within. By selecting species through this catalogue, you are transferred to the species-specific page.

Every species-specific page includes a characteristic photo of the species, its common name in Greek and English, the official Latin name, taxonomic information, physical description, basic biology information, notes on local and global (if applicable) distribution, conservation status and the species occurrence map.

The species occurrence map is developed on a 5 km x 5 km grid where red grid cells indicate confirmed occurrence of the species in that cell. The map has a simple grey background of the island and is openly available for download in kmz format.

**Contact information**: The page has a short text on the importance of citizen science in national-wide research and notes the basic information required with each observation (e.g. species name or photo, location, date). It also highlights the means of communication with the scientific team of the Atlas. These are via e-mail (herpatlascy@gmail.com), through a phone number (for Viber and WhatsApp messages) and through social media (Facebook, Twitter and Instagram).

**Sources**: The Cyprus Herp Atlas collects and centralises information from various sources. Here, we present the number of observations per source type as mentioned on the Database. For scientific journals, books and reports, the formal reference is also visible.

**The team**: Α brief page containing basic information about the Open University of Cyprus and the Herpetological Society of Cyprus that are maintaining the Cyprus Herp Atlas initiative and links to their own websites.

## Figures and Tables

**Table 1.** Detailed information of the Data and Metadata categories included within the Cyprus Herp Atlas database.

DATA	
**ID**:	A number that corresponds to a singular observation and can act as a reference point for it.
**Species**:	The official scientific name of the species.
**Longitude**:	The longitude coordinate, in the World Geodetical System (WGS84).
**Latitude**:	The latitude coordinate, in the World Geodetical System (WGS84).
METADATA	
**Person**:	The name of the person or organisation that provided the observation.
**Contact**:	The contact information (e.g. mobile number or e-mail address) of the person who provided the observation in case clarification or further details are required. For organisations or public authorities, the contact information corresponds to the officer who is handling the data.
**Source**:	The source of the observation. Currently four sources are being applied (i.e. books, citizen science, scientific literature, public departments). A fifth category (incomplete) has been used in cases where critical metadata information is missing or totally absent.
**Sub-source**:	Based on the source, various sub-sources are applied directing - for example - to the name of the Public Department or the specific social media, from where data were retrieved.
**Ownership**:	The name of the person or organisation the data belong to. In cases of journals, reports etc., the formal reference is used.
**Date**:	The exact date when the observation was made. In some cases, when the exact date is not available, the month and/or year are noted.
**FileID**:	An abbreviation that corresponds to the name of the file where the raw data of each observation are stored.
**Roadkill**:	This is a binary field (Yes/No) and corresponds to the circumstances during the observation. “Yes” if the animal was recorded dead on the road.
**Individuals**:	In some cases, more than one individual of a species is observed, but recorded as a single observation. In these cases, the total number of individuals can be noted in this column.
**Comments**:	Any comments that will not fit into any of the other fields, but might be important (e.g. species morph or health condition).
